# An Analysis of Individual Differences in Within-Test Practice Effects in Progressive Matrices

**DOI:** 10.3390/jintelligence13110147

**Published:** 2025-11-13

**Authors:** José H. Lozano, Susan E. Embretson, Javier Revuelta

**Affiliations:** 1 Department of Social Psychology and Methodology, Faculty of Psychology, Universidad Autónoma de Madrid, 28049 Madrid, Spain; javier.revuelta@uam.es; 2 School of Psychology, Georgia Institute of Technology, Atlanta, GA 30332, USA; susan.embretson@psych.gatech.edu

**Keywords:** progressive matrices, non-verbal reasoning, practice effect, person-specific effects, operation-specific learning, linear logistic test model

## Abstract

The present study aimed to investigate individual differences in practice effects during progressive matrices based on Carpenter et al.’s taxonomy of abstract rules. To this end, data from a non-verbal reasoning test, the Abstract Reasoning Test (ART), were used. Because the ART was developed from Carpenter et al.’s theory, the impact of extraneous factors unrelated to the theoretical model is minimized, thereby allowing for a more precise identification of practice effects. The sample consisted of 765 military recruits who responded to 34 items on the ART. Analyses were conducted using a random weights operation-specific learning model (RWOSLM), in which practice parameters were treated as random effects allowed to vary across individuals. The model measures within-test practice effects specific to each examinee, allowing the hypothesis of rule learning during the ART to be assessed at the individual level. Correlations between practice effects and external measures associated with intelligence were examined to investigate the nature of the practice effects. The results suggest individual differences in rule learning within the ART. Decreases in difficulty were observed for both pairwise progression and figure addition or subtraction, although between-person variability was evident only for the latter. Additionally, the results revealed between-person variability in decreases in difficulty associated with one of the items’ figural properties, which suggests the existence of individual differences in the process of increasing familiarity with this feature throughout the test. Individual differences in practice effects during the ART significantly correlated with external measures of abilities and intellect, suggesting that practice effects during progressive matrices are conceptually tied to intelligence.

## 1. Introduction

Identifying the cognitive processes underlying performance on intelligence tests is essential for developing a comprehensive understanding of the nature of intelligence. However, the inherent complexity of intelligence tests often makes this identification challenging, thereby perpetuating ongoing debates about the definition and scope of intelligence. To elucidate these processes, rigorous experimental and psychometric analyses of the most representative measures of intelligence are required.

One of the most influential and studied intelligence tests is the Raven’s Advanced Progressive Matrices (RAPM; J. C. Raven 1965). Originally developed by John C. Raven, the progressive matrices were designed to assess the ability to educe relations and correlates, aligning with Spearman’s (1927) definition of the general intelligence factor (g). The RAPM is a derivative of the original progressive matrices designed to spread the scores of the top 20% of the population (Raven and Raven 2003). The centrality of the RAPM among cognitive ability tests has led many experts to consider it the best measure of general intelligence (e.g., Jensen 1998; Snow et al. 1984). Each RAPM item consists of a 3 × 3 matrix of figural elements with the bottom right entry missing. The elements in the matrix follow a pattern based on abstract rules, so respondents must induce the rules in order to identify the element that fills the missing entry.

Based on the analysis of verbal protocols, eye fixation patterns, and errors, Carpenter et al. (1990) developed a processing theory of performance on the RAPM. The authors identified five analytic rules that govern the elements in the matrix (Carpenter et al. 1990): constant in a row (CR: the same value occurs throughout a row, but changes down a column); quantitative pairwise progression (PP: a quantitative increment or decrement occurs between adjacent entries in an attribute such as size, position, or number); figure addition or subtraction (A/S: a figure from one column is added to or subtracted from another figure to produce the third); the distribution of three values (D3: three values from a categorical attribute are distributed through a row); and the distribution of two values (D2: two values from a categorical attribute are distributed through a row; the third value is null). The theory postulates two processes involved in solving RAPM items: correspondence finding and goal management. Correspondence finding entails identifying the elements in the matrix that are governed by a rule, whereas goal management involves decomposing the problem into sub-goals in order to facilitate exploring tentative solution paths while preserving previous progress. These processes rely on different abilities and are affected by different item features. Specifically, correspondence finding relies on the ability to induce abstract relations and is influenced by the type of rule, whereas goal management relies on working memory and is influenced by the number of rules involved in the item. As a result, the difficulty of RAPM items depends on the type and number of rules involved in their resolution. According to Carpenter et al. (1990), the easiest rule to induce is CR, which requires storing only one value for an attribute. The induction of PP is also relatively easy, requiring only basic perceptual comparison of two elements for the pairwise relation to be extrapolated to the third. A/S, D3, and D2, in contrast, are more difficult to induce than PP since they involve conceptual processes and require simultaneous consideration of all three elements. Finally, D2 is the most difficult rule to induce as it requires the additional ability to form abstract correspondences involving null arguments.

In addition, subsequent research has shown that the drawing features involved in the matrices may constitute another source of difficulty in the RAPM (Embretson 2002). More specifically, Embretson (2002) proposed a classification of three figural properties that may impact performance on the RAPM: overlay, distortion, and fusion. Overlay occurs when multiple objects in a matrix entry are superimposed, distortion occurs when the shapes of corresponding objects are deformed, and fusion occurs when multiple objects in an entry can be perceived as one larger object. Embretson (2002) found that distortion was positively related to item difficulty in the RAPM, whereas overlay and fusion correlated negatively with item difficulty, although the effect of overlay was only marginally significant.

Despite the consensus on the relevance of Carpenter et al.’s (1990) theory, later studies have suggested that learning processes may also occur during the RAPM (e.g., Birney et al. 2017; Bui and Birney 2014; Ren et al. 2014; Verguts and De Boeck 2002). These processes unfold across items as individuals practice the rules required to solve them. The hypothesis of learning during the RAPM aligns with Raven’s own conception of the progressive matrices, as he acknowledged that certain learning processes took place while responding to the items (J. Raven 2008). Confirming the existence of rule-learning processes in a benchmark intelligence test such as the RAPM would have important theoretical implications, as it would require incorporating learning into the model as a fundamental component of intelligence.

Although the above-mentioned findings challenge the current theoretical framework of RAPM performance, the existing evidence supporting the learning hypothesis remains inconclusive. Most of the evidence was obtained either under conditions more favorable to learning (e.g., increased exposure to items, explicit feedback, etc.) than those involved in the standard administration of the RAPM or by using modified versions of the items (e.g., Bui and Birney 2014; Verguts and De Boeck 2002), which raises concerns about the validity of the measure as an indicator of general intelligence and the generalizability of the results to the standard use of the RAPM. Additionally, other studies have identified an item-position effect during the RAPM (e.g., Lozano 2015; Ren et al. 2014; Schweizer et al. 2009). However, attributing this effect to rule learning requires further empirical validation.

Lozano and Revuelta (2020) investigated practice effects associated with the abstract rules involved in the RAPM under standard administration conditions. To that end, the authors used operation-specific learning models (Fischer and Formann 1982; Lozano and Revuelta 2021; Spada 1977; Spada and McGaw 1985) in combination with Carpenter et al.’s (1990) taxonomy of rules. This approach is particularly suitable for gathering information on the rule-learning hypothesis, as it allows for modeling practice effects specifically associated with each of the abstract rules involved in the test. However, their results did not support the existence of rule learning in the RAPM. On the contrary, all rules showed a significant increase in difficulty throughout the test as a function of practice. Interestingly, the results suggested the presence of learning effects associated with the items’ figural properties, a possibility that had not received attention in the previous literature.

Nevertheless, Lozano and Revuelta’s (2020) study presents certain limitations that warrant consideration. The models used to analyze the data assume that item difficulty is solely determined by a well-defined set of components (in this case, the RAPM abstract rules and figural properties described above), along with the practice effects associated with these components. However, this assumption may be overly restrictive in the case of the RAPM, which was not designed based on a predefined set of rules. In this regard, unaccounted factors within the RAPM may influence item difficulty, contributing to the observed increase in rule difficulty during the test, and potentially obscuring the presence of rule-learning effects. Consequently, the application of operation-specific learning models to data from progressive matrices strictly generated based on Carpenter et al.’s (1990) taxonomy may help to circumvent this issue and provide complementary information regarding the learning hypothesis in the RAPM.

## 2. The Present Study

The present study aims to investigate within-test practice effects in theoretically generated progressive matrices based on Carpenter et al.’s (1990) model. This approach minimizes the impact of factors not included in the theoretical model on the study of practice effects. Consequently, changes in difficulty during the test associated with specific components can be more accurately attributed to practice effects such as learning or fatigue. Specifically, the present study analyzed data from the Abstract Reasoning Test (ART; Embretson 1998). The ART was developed using a cognitive design system approach, a method that integrates cognitive theory into test construction to ensure validity. The ART was specifically developed from Carpenter et al.’s (1990) cognitive theory, and its item structures were originally conceived to match the structures studied by Carpenter et al. (1990). The ART’s construct validity has been strongly supported through multiple studies. Specifically, the ART version used in the present study showed a correlation of .78 with the RAPM (Embretson 1998, 2002). In the present study, the analysis of practice effects within the ART was conducted using a random weights operation-specific learning model (RWOSLM; Lozano and Revuelta 2023). The RWOSLM measures within-test practice effects specific to each examinee, which may provide relevant information regarding rule learning during the ART progressive matrices at the individual level. Additionally, correlations between practice effects and external measures associated with intelligence were examined to investigate the nature of the practice effects.

## 3. Materials and Methods

### 3.1. Research Model and Statistical Analyses

The RWOSLM is a random weights linear logistic test model (Rijmen and De Boeck 2002) aimed to measure individual differences in operation-specific practice effects. According to the RWOSLM, the logit of the success probability of person *i* (i=1,…,n) on item *j* (j=1,…,J) is given by

(1) $$\mathrm{logit}\left[P\left(X_{ij}=1\right)\right]=\theta_{i}-\sum_{m=1}^{M}w_{jm}\alpha_{m}+\sum_{m=1}^{M}v_{jm}\delta_{im},$$

where:

θ*_i_* is the ability of person *i*;

α*_m_* is the difficulty of rule *m* (m=1,…,M);

δ*_im_* is the effect of previous practice on the difficulty of rule *m* for person *i*;

*w_jm_* is the weight of rule *m* on item *j*;

*v_jm_* is given by

(2) $$v_{jm}=w_{jm}\sum_{k=1}^{j-1}w_{km},$$

where *w_km_* is the weight of rule *m* on the previous item *k* (k=1,…,j−1).

The model also includes **W**, a *J* × *M* matrix that contains the weights *w_jm_*, and **V**, a *J* × *M* matrix that contains the weights *v_jm_*. The weights *w_jm_* and *v_jm_* are not estimated. Instead, they are part of the hypothesized test structure, which must be specified before the model is applied.

In this context, the ability parameter θ*_i_* represents abstract reasoning; that is, the ability of person *i* to identify and apply the rules involved in the item regardless of practice effects. The difficulty parameter α*_m_* represents the initial difficulty (without practice) of rule *m*. The practice parameter δ*_im_* represents the effect of practicing rule *m* for person *i*. A positive sign for the parameter δ*_im_* indicates a decrease in the difficulty of operation *m* for person *i* as a function of practice, which may be interpreted as learning. On the other hand, a negative sign for the parameter δ*_im_* indicates an increase in the difficulty of operation *m* for person *i* as a function of practice, which may be interpreted as fatigue or loss of interest and/or attention. In this regard, the practice effect may be conceived as a change in the difficulty of a rule for a particular examinee as a function of practice:

(3) $$\alpha_{ijm}=\alpha_{m}-\sum_{k=1}^{j-1}w_{km}\delta_{im},$$

where α*_ijm_* is the difficulty of rule *m* for person *i* at item *j*.

For the model to be identified, the matrices **W** and **V**^+^ = (**V**, **1**) (i.e., **V** supplemented with a column vector of ones) and the concatenation of both matrices must have a full column rank. Additionally, the number of effects should not exceed the number of items (i.e., 2*M* + 1 ≤ *J*)^1^ and the covariance matrix of the practice effects must be symmetric positive definite.

Model estimation and evaluation were based on a Bayesian framework. The RWOSLM (Lozano and Revuelta 2023) was compared with other models: the operation-specific learning model (OSLM; Fischer and Formann 1982; Lozano and Revuelta 2021; Scheiblechner 1972; Spada 1977; Spada and McGaw 1985), the linear logistic test model (LLTM; Fischer 1973; Scheiblechner 1972), and the random weights linear logistic test model (RWLLTM; Rijmen and De Boeck 2002). While in the RWOSLM the difficulty parameters (α*_m_*) are constant across individuals and the practice parameters (δ*_im_*) are random across individuals^2^, in the OSLM both difficulty (α*_m_*) and practice (δ*_m_*) parameters are constant across individuals. In this regard, the OSLM represents the absence of individual differences in practice effects. Additionally, the LLTM and the RWLLTM explain item difficulty exclusively as a function of the difficulty of the abstract rules involved in the item. Therefore, both models represent the absence of within-test practice effects. The difference between these two models resides in that the LLTM treats the difficulty parameters as fixed effects constant across individuals (from now on denoted as η*_m_*), whereas the RWLLTM treats them as random effects varying across individuals (from now on denoted as η*_im_*).

Based on previous research (Lozano and Revuelta 2023), the prior distributions for the RWOSLM parameters were

(4) $$\theta_{i}∼N\left(0,\;1\right),$$

(5) $$\alpha_{m}∼N\left(0,\;100\right),\;\mathrm{and}$$

(6) $$\delta_{i}∼N\left(\mu_{\delta },\;\Sigma_{\delta }\right),$$

where $\delta_{i}=\left(\delta_{i1},\ldots ,\delta_{iM}\right)$ is an *M* vector of practice parameters of person *i*; $\mu_{\delta }=\left(\mu_{\delta_{1}},\ldots ,\mu_{\delta_{M}}\right)$ is an *M* prior mean vector; $\Sigma_{\delta }=\mathrm{diag}\left(\sigma_{\delta }\right)\Omega_{\delta }\mathrm{diag}\left(\sigma_{\delta }\right)$ is an *M* × *M* prior covariance matrix; $\sigma_{\delta }=\left(\sigma_{\delta_{1}},\ldots ,\sigma_{\delta_{M}}\right)$ is an *M* vector of prior standard deviations; and $\Omega_{\delta }$ is a prior correlation matrix.

The hyper-prior distributions for the hyper-parameters were

(7) $$\mu_{\delta_{m}}∼N\left(0,\;1\right),$$

(8) $$\sigma_{\delta_{m}}∼\text{half-Cauchy}\left(0,\;5\right),\;\mathrm{and}$$

(9) $$\Omega_{\delta }∼\mathrm{LKJ}\left(2\right),$$

where LKJ is the distribution developed by Lewandowski et al. (2009), which is less informative than the Wishart prior traditionally used for covariance matrices. Note that the prior standard deviations of the practice parameters ($\sigma_{\delta_{m}}$) are informative about individual differences in practice effects.

Additionally, based on previous research (Lozano and Revuelta 2021, 2023), an *N*(0, 100) prior distribution was specified for the fixed-effects parameters of the OSLM (α*_m_* and δ*_m_*). In line with the previous specifications, the random-effects operation difficulty parameter of the RWLLTM (η*_im_*) was assumed to follow the same multivariate normal prior distribution specified for δ*_im_* in the RWOSLM, whereas the fixed-effects operation difficulty parameter of the LLTM (η*_m_*) was assumed to follow an *N*(0, 100) prior distribution. A standard normal prior distribution was specified for the person ability parameter (θ*_i_*) in all models.

Two information criterion measures were used for model comparison and selection: the widely applicable information criteria (WAIC; Watanabe 2010, 2013) and the leave-one-out information criterion (LOOIC; Gelman et al. 2014; Vehtari et al. 2017). These measures quantify the discrepancy between the model and the data while penalizing for model complexity. This penalty compensates for the over-fitting exhibited by more complex models as a result of their greater flexibility. Lower values indicate a better balance between fit and parsimony. Both WAIC and LOOIC have proven useful for detecting within-test practice effects (Lozano and Revuelta 2021, 2023).

Complementarily, posterior predictive model checking (PPMC; Gelman et al. 1996) was used to assess the fit of the models to the data. The posterior predictive *p*-value (*p*_post_; Meng 1994) was computed using the odds ratio (OR; Chen and Thissen 1997; Sinharay 2005) as a test statistic. The OR statistic is a measure of association between pairs of items that have been effective in identifying within-test practice effects (Lozano and Revuelta 2021, 2023).

### 3.2. Software

The analyses were conducted with R version 4.4.0 (R Core Team 2024) and the RStan R package version 2.35 (Stan Development Team 2024). Markov chain Monte Carlo simulation was performed using the No-U-Turn Sampler (NUTS; Hoffman and Gelman 2014). Four Markov chains of 5000 samples each were run. The first half of the samples were discarded as burn-in. The potential scale reduction statistic ($\hat{R}$; Gelman and Rubin 1992) was used to evaluate the convergence of the chains. WAIC and LOOIC were obtained using the loo R package version 2.8.0 (Vehtari et al. 2024).

### 3.3. Measures and Data

**Figure 1:**
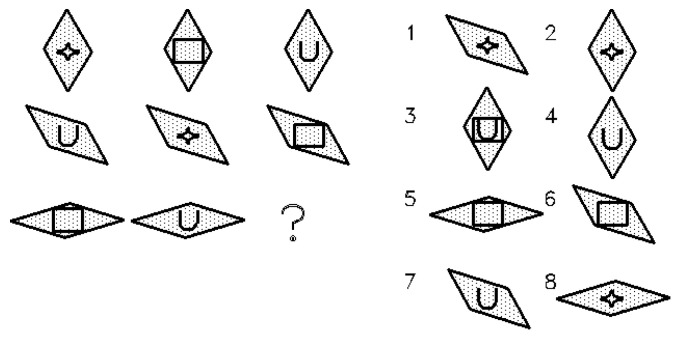
Example of ART item.

The present study is based on data originally collected by Embretson (1998). The dataset comprised responses of 818 military recruits (80% males) to 34 items of the ART. Participants were randomly assigned to three groups which were administered equivalent computerized forms of the ART. The items administered to the three groups were generated from the same theoretical structure (same number and type of rules) and figural properties, although the specific objects varied between groups. Each form included thirty structurally matched items and four anchor items distributed across the test. Items were ordered by predicted difficulty and their position was equated across forms. Appendix A shows the theoretical structure of the three test forms. Figure 1 presents an example of an ART item involving CR, D3, and overlay; the correct answer is 8. The matrices **W** and **V**^+^, and the concatenation of both matrices, satisfy the full column rank condition, and the number of effects does not exceed the number of items.

A total of 6.5% of participants were excluded due to response latencies less than one second, which were interpreted as involuntary responses resulting from the computerized administration of the items. The final group sample size was *n* = 765. A linear regression analysis showed that abstract rules and figural properties accounted for 70% of the variance in item difficulty, operationalized as Rasch item difficulty parameters. Item position did not significantly contribute to the explanation of item difficulty beyond the abstract rules and figural properties.

In addition to the ART, participants completed several other measures as part of the original study. For the present study, we utilized a General Test Battery (GTB) and the Revised NEO Personality Inventory (NEO PI-R; Costa and McCrae 1992) to examine their relationships with individual differences in practice effects in the ART. GTB aggregates several measures of cognitive abilities and exhibits a high load on the general factor of intelligence. The correlation between GTB and the RAPM is .48 (Embretson 1998). Regarding the NEO PI-R, previous research suggests that intelligence is positively and moderately related to Openness, while marginal and mostly inconsistent relationships have been observed with the other personality dimensions (DeYoung 2020).

## 4. Results

Table 1 shows the goodness-of-fit estimates for the fitted models. The RWOSLM yielded the lowest WAIC and LOOIC values, indicating superior out-of-sample predictive performance. However, an inspection of the prior variances associated with the RWOSLM practice parameters revealed that only A/S and distortion exhibited substantial variability in practice effects. Subsequently, a constrained version of the RWOSLM (hereafter referred to as RWOSLM_const._) was fitted to the data in which all practice parameters, except those associated with A/S and distortion, were treated as fixed effects. The RWOSLM_const._ retained the lowest WAIC and LOOIC values among all models. Although posterior predictive checks indicated that none of the models showed a good fit to the data (all *p*_post_ < .05), the RWOSLM_const._ generated replicated OR values that more closely approximated the realized values, suggesting that this model was the most appropriate for analyzing within-test practice effects in the ART.

Table 2 shows the expected a posteriori (EAP) estimates, posterior standard deviations, and posterior probability intervals for the RWOSLM_const._ parameters. The positive sign of the EAP estimate of the practice parameter associated with PP (δ_PP_), together with the absence of zero within the posterior probability interval, suggested a decrease in the difficulty of this rule throughout the test. In the case of D3 and distortion, zero was included in the posterior probability interval of the practice parameter (δ_D3_) and in the posterior probability interval of the prior mean of the practice parameter ($\mu_{\delta_{\mathrm{Distortion}}}$), respectively, indicating a low posterior probability of a decrease in difficulty throughout the test: *P*(δ_D3_ > 0 | ***x***) = .196 and *P*($\mu_{\delta_{\mathrm{Distortion}}}$> 0 | ***x***) = .399. Conversely, the negative signs of the EAP estimates of the practice parameters associated with CR, D2, overlay, and fusion (δ_CR_, δ_D2_, δ_Overlay_, and δ_Fusion_) and the negative sign of the EAP estimate of the prior mean of the practice parameter associated with A/S ($\mu_{\delta_{A/S}}$), together with the absence of zero within the corresponding posterior probability intervals, suggested an increase in difficulty for these components throughout the test.

**Figure 2:**
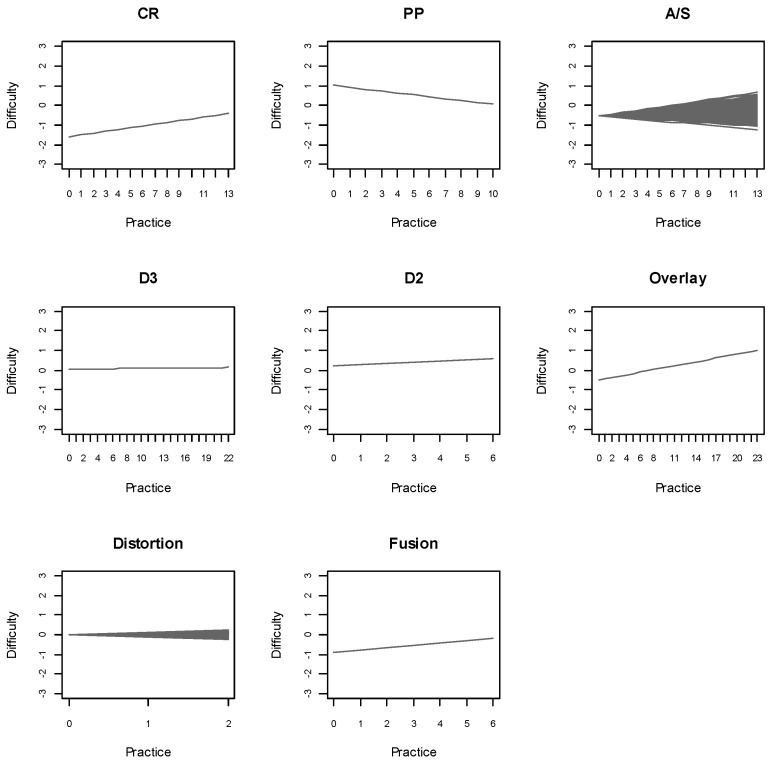
Difficulty of the ART rules and figural properties as a function of practice according to the RWOSLM_const._.

The previous results represent overall trends. However, the estimated prior variances of the practice parameters of A/S and distortion ($\sigma_{\delta_{A/S}}^{2}$ and $\sigma_{\delta_{\mathrm{Distortion}}}^{2}$) indicated the presence of inter-individual variability in the practice effects associated with these components. Figure 2 shows the difficulty of the ART rules and figural properties as a function of practice, revealing the presence of individual differences in the practice effects associated with A/S and distortion. An analysis at the individual level revealed that 19% and 37% of the participants exhibited a positive EAP estimate of the practice parameter associated with A/S (δ*_i_*_A/S_) and distortion (δ*_i_*_Distortion_), respectively, suggesting an operation-specific reduction in difficulty throughout the test for the respective proportions of participants.

Table 3 shows the Pearson correlations between the EAP estimates of the random-effects parameters of the RWOSLM_const._ and the reference measures GTB and NEO PI-R. As expected, the ability parameter (θ*_i_*) correlated positively and significantly with GTB and Openness. It also correlated negatively and significantly with Conscientiousness, and showed marginal negative correlations with Agreeableness and Neuroticism. With regard to the practice parameters, the practice parameter associated with A/S correlated positively and significantly with GTB, negatively and significantly with Conscientiousness and Agreeableness, and exhibited a marginal positive correlation with Openness; whereas the practice parameter associated with distortion correlated positively and significantly with GTB and Openness, and showed marginal negative correlations with Conscientiousness, Extraversion, and Agreeableness.

## 5. Discussion

The conceptualization of intelligence has historically lagged behind its measurement. Understanding the cognitive processes underlying performance on intelligence tests has played a crucial role in shaping our definition of intelligence. Consequently, identifying individual differences in learning processes when using reference measurement instruments could significantly influence our conceptualization of intelligence. In this vein, this study aimed to examine inter-individual variability in the practice effects associated with the abstract rules involved in the ART. The fact that the ART was developed based on Carpenter et al.’s (1990) theory minimizes the impact of extraneous factors unrelated to the theoretical model, thereby allowing for a more accurate identification of practice effects.

Evidence of decreases in difficulty associated with abstract rules during the ART was scarce, primarily observed in PP and, to a lesser extent, in A/S. The evidence concerning PP was consistent across participants, suggesting that performance improvements in this rule occur naturally throughout the test. In contrast, evidence related to A/S was far more limited, indicating that improvements with this rule are considerably more challenging to achieve. Specifically, only 19% of participants showed a decrease in difficulty associated with A/S. Individual differences in practice effects associated with A/S correlated positively with the reference ability measure and Openness. These findings suggest that practice effects during the ART are fundamentally linked to intelligence, as they predict performance on complex ability tasks and relate to measures of intellect.

Regarding the items’ figural properties, a decrease in difficulty throughout the test was observed only for distortion. Specifically, 37% of participants showed a reduction in difficulty, suggesting the presence of learning effects related to this property. The positive and significant correlations with GTB and Openness further support the association between individual differences in practice effects within the ART and intelligence.

The increases in difficulty observed in CR, D2, overlay, and fusion, as well as in A/S and distortion for most participants, may be interpreted as progressive fatigue or a decline in interest and/or attention throughout the test. The results therefore suggest that participants improve performance on some components and exhibit impairments on others, both as a result of practice. Alternatively, an increasing presence of difficulty factors not considered by the model as the test progresses could also explain the rising difficulty observed for some rules. However, the fact that the ART was generated directly from theory minimizes the risk of unmodeled factors. Moreover, the results indicated that item position did not significantly contribute to explaining item difficulty beyond what the model’s components accounted for, which provides no evidence for extraneous factors accounting for the observed increases in difficulty.

Nevertheless, it is important to add two cautionary notes to the discussion. First, the additive nature of the fitted models in regard to item difficulty ignored the possibility of interaction effects between components. In this sense, an observed increase in rule difficulty throughout the test might be partially due to interaction effects between rules. Although a linear modeling approach to performance on the ART may be efficient (see also Embretson 1998), the possibility of interaction effects cannot be dismissed. The RWOSLM allows specifying interactions between operations by including new components in the structure matrix that represent the combination of two or more operations within the same item. However, in the case of the ART, the high number of components in the concatenation of the matrices **W** and **V** makes it difficult to explore these potential interactions without the estimates becoming highly unstable. Future research on operation-specific learning modeling may help address this issue. Second, the linearity of the fitted models assumed that the relationship between rule difficulty and practice is constant throughout the entire test. However, the literature on learning and fatigue has traditionally suggested that these processes are better represented by curves. In this regard, future studies may be aimed at investigating nonlinear practice effects within progressive matrices.

In summary, this study provides evidence of inter-individual variability in within-test practice effects during the ART. Although evidence supporting learning effects during the test was limited to only a few components of the structure matrix, the results provide relevant information regarding the cognitive processes involved in solving progressive matrices. Given the centrality of progressive matrices in the measurement of intelligence, it would be valuable to investigate whether similar effects occur in other inductive reasoning tests based on pattern recognition in figural matrices. Future research using advanced dynamic item response models could further explore this possibility.

## Figures and Tables

**Table 1:** Goodness-of-fit estimates for the fitted models.

	WAIC				LOO				OR		
Model	elpd_waic_	*p*_waic_	waic		elpd_loo_	*p*_loo_	looic		Observed	Simulated (Sd)	*p*_post_
RWOSLM	−14,320.8	1060.8	28,641.7		−14,330.5	1070.5	28,661.0		1344.92	1112.31 (30.40)	.000
RWOSLM_const._	−14,326.0	848.2	28,652.0		−14,331.5	853.6	28,662.9		1344.92	1118.90 (29.45)	.000
OSLM	−14,339.8	653.9	28,679.5		−14,341.4	655.5	28,682.8		1344.92	1117.81 (30.13)	.000
RWLLTM	−14,805.8	1052.0	29,611.7		−14,812.6	1058.7	29,625.2		1344.92	1064.48 (25.69)	.000
LLTM	−14,814.3	646.8	29,628.6		−14,816.1	648.6	29,632.3		1344.92	1059.84 (25.65)	.000

**Table 2:** Expected a posteriori estimates (EAP), posterior standard deviations (SD), and posterior probability intervals (2.5–97.5%) for the RWOSLM_const._ parameters.

	EAP	SD	2.5%	97.5%		EAP	SD	2.5%	97.5%		EAP	SD	2.5%	97.5%
α_CR_	−1.585	0.103	−1.784	−1.391	δ_CR_	−0.090	0.011	−0.112	−0.068					
α_PP_	1.017	0.069	0.892	1.156	δ_PP_	0.095	0.010	0.075	0.115					
α_A/S_	−0.538	0.072	−0.674	−0.399	$\mu_{\delta_{A/S}}$	−0.022	0.009	−0.040	−0.004	$\sigma_{\delta_{A/S}}^{2}$	0.003	0.001	0.001	0.004
α_D3_	0.045	0.064	−0.080	0.171	δ_D3_	−0.004	0.005	−0.014	0.006					
α_D2_	0.213	0.045	0.122	0.305	δ_D2_	−0.060	0.015	−0.089	−0.030					
α_Overlay_	−0.503	0.106	−0.719	−0.290	δ_Overlay_	−0.066	0.008	−0.080	−0.051					
α_Distortion_	−0.004	0.092	−0.184	0.176	$\mu_{\delta_{\mathrm{Distortion}}}$	−0.017	0.075	−0.168	0.136	$\sigma_{\delta_{\mathrm{Distortion}}}^{2}$	0.042	0.011	0.001	0.144
α_Fusion_	−0.906	0.122	−1.138	−0.673	δ_Fusion_	−0.125	0.034	−0.191	−0.059					

**Table 3:** Pearson correlations between the EAP estimates of the random-effects practice parameters of the RWOSLM_const._ and the reference measures.

	θ*_i_*	δ*_i_*_A/S_	δ*_i_*_Distortion_
GTB	.513 **	.254 **	.221 **
Openness	.175 **	.075 *	.097 **
Conscientiousness	−.098 **	−.103 **	−.079 *
Extraversion	.014	−.065	−.085 *
Agreeableness	−.076 *	−.094 **	−.091 *
Neuroticism	−.071 *	−.040	−.005

** significant at the .01 level (two-tailed); * significant at the .05 level (two-tailed).

## Data Availability

No new data were created or analyzed in this study. Data sharing is not applicable to this article.

## Notes

1 In Bayesian inference, this restriction is relaxed to 2*M* ≤ *J* (Lozano and Revuelta 2023).

2 In the Bayesian approach, all model parameters are random variables. The terms constant and random are used here to denote invariability/variability across individuals.

## Appendix A

**Table A1:** Transposed **W** matrix of the ART.

Items	CR	PP	A/S	D3	D2	O	D	F
1	1	2	0	0	0	0	0	0
2	1	0	0	1	0	1	0	0
3	0	0	0	2	0	1	0	0
4	1	1	0	1	0	0	0	0
5	1	0	0	0	0	1	0	1
6	0	0	1	0	0	1	0	0
7	1	0	0	2	0	1	0	0
8	0	0	0	2	0	1	0	0
9	1	1	0	0	0	1	0	1
10	1	1	0	0	0	1	0	1
11	1	1	0	0	0	0	0	0
12	0	0	1	0	0	1	0	1
13	0	0	0	2	0	1	0	0
14	0	0	1	0	0	0	0	1
15	0	0	1	0	0	0	0	1
16	0	0	1	0	0	1	0	0
17	1	1	0	1	0	1	0	0
18	0	0	1	0	0	1	0	0
19	1	1	0	0	0	0	0	0
20	0	0	2	0	0	1	0	0
21	0	0	1	0	0	1	0	0
22	1	0	0	2	0	0	0	0
23	0	0	1	0	0	1	0	0
24	0	0	1	0	1	1	0	0
25	1	2	0	0	0	1	0	1
26	0	0	1	0	0	1	0	0
27	0	0	1	0	0	1	0	0
28	0	0	1	0	0	0	0	0
29	0	0	0	2	0	0	1	0
30	0	0	0	4	0	1	0	0
31	0	1	0	2	0	0	1	0
32	0	0	0	0	3	1	0	0
33	1	0	0	0	3	1	0	0
34	1	0	0	2	0	1	1	0

CR = constant in a row; PP = quantitative pairwise progression; A/S = figure addition or subtraction; D3 = distribution of three values; D2 = distribution of two values; O = overlay; D = distortion; F = fusion.
